# Comparative effects of a calcium chelator (BAPTA-AM) and melatonin on cryopreservation-induced oxidative stress and damage in ovarian tissue

**DOI:** 10.1038/s41598-023-49892-7

**Published:** 2023-12-21

**Authors:** Atefeh Najafi, Ebrahim Asadi, James D. Benson

**Affiliations:** Department of Biology, University of Saskatchewan, Saskatoon, SK S7N 5E2 Canada

**Keywords:** Cell biology, Quality of life, Paediatric cancer

## Abstract

Oncology treatments cause infertility, and ovarian tissue cryopreservation and transplantation (OTCT) is the only option for fertility preservation in prepubertal girls with cancer. However, OTCT is associated with massive follicle loss. Here, we aimed to determine the effect of supplementation of slow freezing and vitrification media with BAPTA-AM and melatonin alone and in combination on ovarian tissue viability, reactive oxygen species (ROS) levels, total antioxidant capacity (TAC), and follicular morphology and viability. Our results indicated that BAPTA-AM and melatonin can significantly improve ovarian tissue viability and the TAC/ROS ratio and reduce ROS generation in frozen-thawed ovarian tissues in slow freezing and vitrification procedures. BAPTA-AM was also found to be less effective on TAC compared to melatonin in vitrified ovarian tissue. While supplementation of slow freezing and vitrification media with BAPTA-AM and/or melatonin could increase the percentage of morphologically intact follicles in cryopreserved ovarian tissues, the differences were not significant. In conclusion, supplementation of cryopreservation media with BAPTA-AM or melatonin improved the outcome of ovarian tissue cryopreservation in both vitrification and slow freezing methods. Our data provide some insight into the importance of modulating redox balance and intracellular Ca^2+^ levels during ovarian tissue cryopreservation to optimize the current cryopreservation methods.

## Introduction

The development of pediatric cancer treatments during the last decade has significantly decreased subsequent mortality rates^1^. Consequently, the number of young and prepubertal cancer survivors has drastically increased. However, these promising survival rates are associated with the gonadotoxic effects of chemotherapy and radiotherapy^2^ which cause premature ovarian failure (POF), infertility, endocrine dysfunction, menopausal symptoms, subsequent psychological problems, and decreased quality of life in cancer survivors^3^. Since 2019 the American Society for Reproductive Medicine (ASRM) has considered ovarian tissue cryopreservation (OTC) as an acceptable fertility preservation method^4^. Ovarian tissue cryopreservation and transplantation (OTCT) is the only option for prepubertal girls with cancer to preserve fertility^5^. Moreover, it is the most flexible option for fertility preservation in patients who cannot postpone cancer treatments^5^.

The most common methods for OTC are broadly categorized as slow freezing and vitrification^6^. OTCT resulted in approximately 200 live births by slow freezing technique^7^ and 4 live births by the vitrification method^8^. In slow freezing approach, ovarian tissue is slowly frozen in the presence of ice to almost − 140 °C, using a low concentration of cryoprotectant agents (CPAs) and programmable freezers with a cooling rate of approximately 1.5 °C/min^9^. On the other hand, vitrification is characterized by the solidification of the sample and surrounding solution into a transparent glassy form without ice crystallization using high concentrations of CPAs and fast to ultrafast cooling rates (500–5000 °C/min)^10^.

Regardless of the approach, the optimization of both techniques remains crucial to reduce cell death and improve the survival rate of follicles in cryopreserved ovarian tissue^11^. For example, OTC is associated with oxidative stress^12,13^. Oxidative stress can cause follicle loss by triggering of primordial follicle activation and subsequent burnout of the ovarian follicle reserve^14^. In fact, peroxidation of polyunsaturated fatty acids (PUFAs), proteins, and DNA, and subsequent apoptosis activation are underlying mechanisms of damage induced by elevated reactive oxygen species (ROS) generation^15–18^. Moreover, elevated ROS can damage membranes and organelles such as mitochondria. Mitochondria and endoplasmic reticulum (ER) are the main pools of calcium (Ca^2+^) in the cell and play an important role in Ca^2+^ homeostasis, which is critical for cell function such as protein regulation, gene expression, cellular growth, and death^19–22^. Furthermore, ROS can trigger intracellular Ca^2+^ release, and increased Ca^2+^ levels can stimulate ROS production^23^. Cryopreservation-induced damages are not limited to ROS elevation, and there is an intracellular calcium overload during cryopreservation^24–27^. Interestingly, a higher and more constant intracellular Ca^2+^ release in human oocytes was reported in slow freezing compared to vitrification^28^.

Previous studies have demonstrated that the addition of exogenous antioxidants such as melatonin to the cryopreservation media ameliorates some of the negative effects of cryopreservation in reproductive cells and tissues^13,29–32^. Melatonin (N-acetyl-5-methoxy tryptamine), an extremely lipophilic molecule, can easily cross cell membranes^33^. While melatonin is mostly made and secreted by the pineal gland^33^, it is probably also produced in the ovary^34^. We recently found that exogenous melatonin can expand the osmotic tolerance limits (OTLs) of the human and bovine ovarian stromal cells and reduces the detrimental effect of osmotic stress by decreasing ROS levels, improving total antioxidant capacity (TAC) and mitochondrial respiratory chain activity^35^.

On the other hand, a previous study indicated that supplementation of vitrification solution with calcium chelators such as ethylene glycol tetra acetic acid (EGTA) and1,2-bis (o-amino phenoxy) ethane-*N*,*N*,*N*′,*N*′-tetra acetic acid (BAPTA-AM) resulted in reduction of cytoplasmic calcium level, preservation of normal mitochondrial function and membrane potential levels and higher survival rates in vitrified oocytes^36,37^. BAPTA-AM is a permeable intracellular calcium ion chelator that binds with two calcium ions via four carboxylic acid groups^38^. A combination of BAPTA and ruthenium red can reduce Ca^2+^ concentration, preserve the natural distribution of cortical granulosa, and improve the fertilization and cleavage rate of the frozen-thawed bovine oocytes after IVF^27^.

Despite the crucial role of calcium in oocyte maturation^39^, and the imbalance of calcium hemostasis during cryopreservation, to the best of our knowledge, there is no study to evaluate the effect of calcium chelators on ovarian tissue cryopreservation. Thus, here we evaluated the effect of supplementation of slow freezing and vitrification media with BAPTA-AM, melatonin, and BAPTA-AM plus melatonin on post-warming cell viability, ROS levels, TAC, follicular viability, and morphology in ovarian tissue. Also, we examined the TAC/ROS ratio in the prediction of ovarian tissue cells and follicles viability during OTC. In the present study bovine ovaries were used as a model for human ovaries because bovine ovaries exhibit many characteristics of human ovaries including the size, the structure, the number of follicles development per/cycle, and molecular pathways and hormonal signaling mechanisms^40–43^.

## Results

### Ovarian tissue cells viability

**Figure 1:**
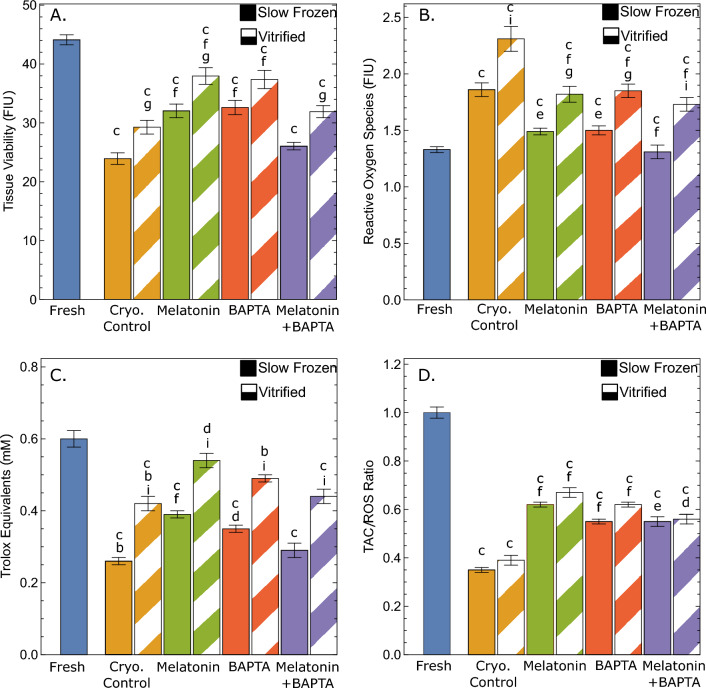
Ovarian tissue viability (**A**), reactive oxygen species (ROS) levels (**B**), total antioxidant capacity (TAC) (**C**), and TAC/ROS ratio (**D**) in BAPTA-AM, melatonin, and BAPTA-AM plus melatonin supplemented groups vs fresh and cryopreservation controls under vitrification and slow freezing protocols. Significance is indicated as follows: b: *p* ≤ 0.01 vs. fresh group; c: *p* ≤ 0.001 vs. fresh group; d: *p* < 0.05 vs. slow freezing or vitrification groups; e: *p* ≤ 0.01 vs. slow freezing or vitrification groups; f: *p* ≤ 0.001 vs. slow freezing or vitrification groups; g: *p* < 0.05 vs. counterpart group; i: *p* ≤ 0.001 vs. counterpart group.

The results of ovarian tissue cells viability (as a fluorescent intensity unit (FIU) ± SEM) in the fresh and cryopreserved groups and their relevant *p* values, are shown in Fig. 1A. Ovarian tissue cells viability in both the slow freezing and vitrification groups was significantly reduced to 23.5 ± 0.97, *p* < 0.001, and 28.8 ± 1.1, *p* < 0.001 compared to its level in the fresh groups (41.4 ± 4.9). In the slow freezing method, ovarian tissue cells viability significantly increased to 31.5 ± 1.1, *p* < 001 and 32.1 ± 1.2, *p* < 001 in cryopreserved groups supplemented with melatonin and BAPTA-AM, respectively compared to the slow freezing control group. Ovarian tissue cells viability was also significantly higher in vitrified groups supplemented with melatonin (37.4 ± 1.3) or BAPTA-AM (36.8 ± 1.5) compared to the vitrified control group (*p* < 0.001). Supplementation of cryopreservation media with a combination of melatonin and BAPTA-AM improved the level of cells viability in slow freezing (25.6 ± 0.63, *p* = 0.93) and vitrification (31.4 ± 1, *p* = 0.81) groups compared to control freezing groups, but these differences were not significant in both freezing techniques. Lastly, ovarian tissue cells viability was significantly higher in vitrified control compared to its counterpart group in the slow freezing technique (*p* = 0.043). Ovarian tissue cells viability was higher in vitrification groups supplemented with melatonin, BAPTA-AM, and melatonin + BAPTA-AM compared to similar groups in slow freezing method.

### Reactive oxidative stress measurement

ROS levels and their related p-values in the fresh and cryopreserved groups are indicated in Fig. 1B. ROS levels (FIU) were dramatically increased in untreated frozen-thawed ovarian cortical pieces in both slow freezing (1.86 ± 0.28, *p* < 0.001) and vitrification (2.31 ± 0.53, *p* < 0.001) groups compared to the fresh group (1.33 ± 0.11). In slow-freezing treatment groups, supplementation of freezing media with melatonin (1.49 ± 0.14, *p* = 0.004), BAPTA-AM (1.50 ± 0.18, *p* = 0.006), and combination of melatonin + BAPTA-AM (1.31 ± 0.28, *p* < 0.001) were significantly reduced ROS levels compared to the slow freezing control group. In vitrification groups, ROS levels were significantly decreased and reached 1.82 ± 0.34 (*p* < 0.001), 1.85 ± 0.28 (*p* < 0.001), and 1.73 ± 0.30 (*p* < 0.001) in melatonin, BAPTA-AM, and melatonin + BAPTA-AM vitrified groups, respectively compared to its level in the control group. Also, ROS levels were significantly higher in vitrification control group compared to their level in slow-freezing control group (*p* < 0.001). The levels of significance between vitrification and slow freezing methods in melatonin treated groups, BAPTA-AM treated groups, and melatonin + BAPTA-AM treated groups were *p* = 0.024, 0.10, and 0.002, respectively.

### Total antioxidant level

The results of the TAC test in all groups and their related *p* value are presented in Fig. 1C. A significant decrease in the frozen-thawed ovarian tissues’ total antioxidant capacity is observed in both slow-freezing (0.26 ± 0.01, *p* < 0.001) and vitrification (0.42 ± 0.02, *p* < 0.001) groups compared to its level in the fresh group, (0.60 ± 0.02). In the slow freezing method, TAC levels were significantly increased in the treated groups with melatonin (0.39 ± 0.02, *p* = 0.001) and BAPTA-AM (0.35 ± 0.01, *p* = 0.041) compared to the slow freezing control group. There was no significant difference between the melatonin + BAPTA-AM treated group compared to slow freezing control group (0.29 ± 0.02, *p* = 0.99). In the vitrification method, TAC levels were significantly increased in the melatonin treated group (0.54 ± 0.02, *p* = 0.048) compared to the vitrification control group. No statistically significant differences were observed in TAC levels between the treated groups with BAPTA-AM (0.49 ± 0.02, *p* = 0.68) and melatonin + BAPTA-AM (0.44 ± 0.01, *p* = 1), and the vitrification control group. Also, significantly higher TAC levels were observed in the vitrification control group compared to their level in the counterpart slow freezing group (*p* < 0.001). TAC levels were significantly higher in the vitrification group supplemented with melatonin (*p* < 0.001), BAPTA-AM (*p* = 0.001), and melatonin + BAPTA-AM (*p* < 0.001) compared to their levels in the counterpart slow freezing groups.

### Follicle viability

**Figure 2:**
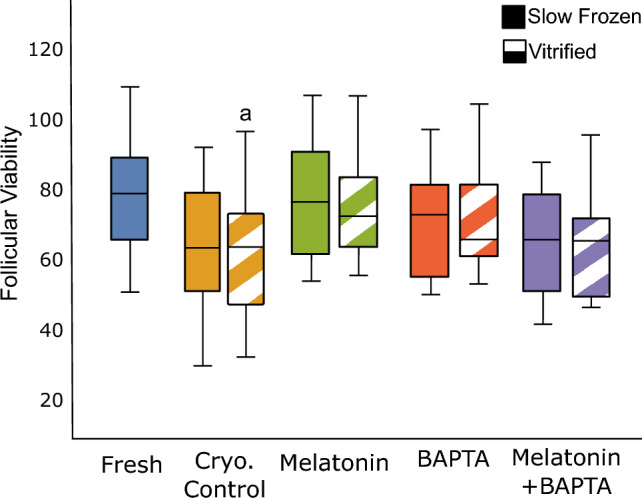
Ovarian follicular viability in BAPTA-AM, melatonin, and BAPTA-AM plus melatonin supplemented groups vs fresh and cryopreservation controls under vitrification and slow freezing protocols. (A). a: *p* < 0.05 vs. fresh group.

**Figure 3:**
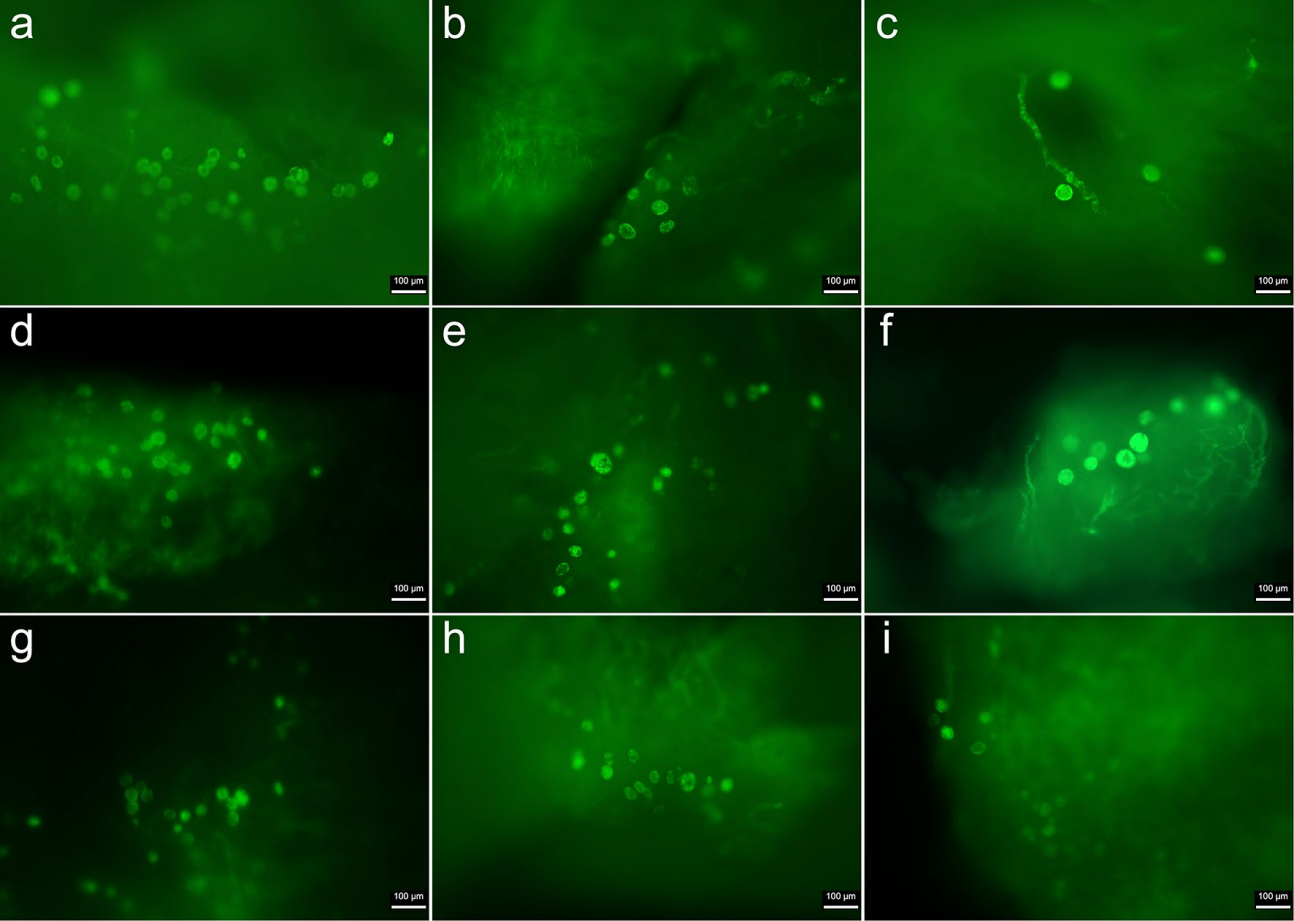
Representative images for follicular viability of ovarian tissue in fresh (**a**), slow freezing control (**b**), vitrification control (**c**), slow freezing melatonin (**d**), vitrification melatonin (**e**), slow freezing BAPTA-AM (**f**), vitrification BAPTA-AM (**g**), slow freezing melatonin plus BAPTA-AM (**h**), and vitrification melatonin plus BAPTA-AM (**i**)—supplemented groups after staining with calcein-AM. Green fluorescence indicates viable follicles. The scale bar represents 100 µm.

The viable follicle count in each group is presented in Fig. 2, and representative images are shown in Fig. 3. The viable follicle count in each group showed an irregular distribution. The viable follicle counts were significantly reduced in the vitrification control group (median 61.75, IQR 43–72) compared to the fresh group (median 78.8, IQR 65.75.–85.87) (*p* = 0.032). There was no significant difference between the number of frozen-thawed ovarian tissue viable follicles in the control slow freezing group (median 64, IQR 50.25–80.5, *p* = 0.25) and the fresh group. Moreover, there was no significant difference observed in the number of viable follicles within the slow freezing and vitrification groups.

### Follicle morphology

**Figure 4:**
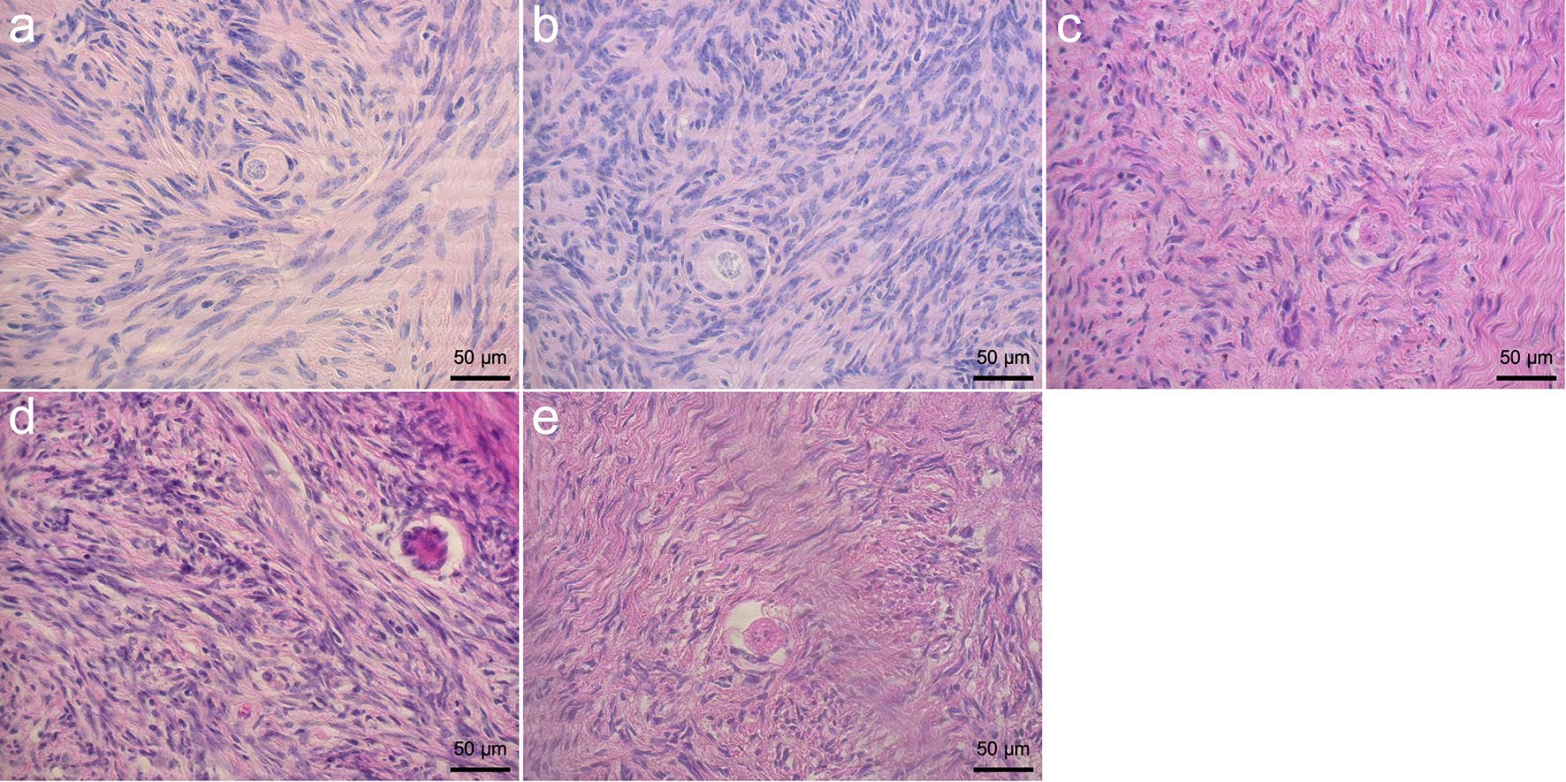
Representative figures for morphologically normal and damaged primordial and primary follicles in ovarian tissue after staining with H & E. Intact primordial (**a**) and primary (**b**), follicles with healthy oocyte and densely compact granulosa cell layers. Damaged follicles with vacuolated oocytes (**c**), abnormal space between the follicle and stroma (**d**), shrunken oocytes (**e**). The scale bar represents 50 um.

A total of 1475 primordial (1107) and primary (368) follicles were examined and categorized as normal and damaged follicles according to their morphology (Fig. 4). Percentages of bovine morphologically normal primordial and primary follicles in ovarian fragments from fresh, and frozen-thawed groups are shown in Table 1. While the percentages of normal bovine primordial and primary follicles significantly decreased from 80.95 ± 1.34 and 62.54 ± 1.56 in the fresh group to 71.45 ± 2.10, *p* < 0.05 and 49.66 ± 2.97, *p* ≤ 0.01 in the control slow freezing and 68.15 ± 2.76, *p* ≤ 0.001 and 51.12 ± 2.87, *p* < 0.05 in the vitrified control groups, there were no significant differences in the percentage of normal primordial and primary follicles between the fresh group and the treatment groups supplemented with melatonin and/or BAPTA-AM, except for the percentage of primary follicles in the slow freezing group supplemented with melatonin plus BAPTA-AM. No difference was observed in the percentage of morphologically normal follicles (primary and primordial) between the slow freezing and vitrification groups. Supplementation of freezing media with melatonin and/or BAPTA-AM increased the percentage of intact follicle compared to control freezing groups whereas this effect was not statistically significant.

**Table 1:** Ovarian follicles morphology in BAPTA-AM, melatonin, and BAPTA-AM plus melatonin supplemented groups vs fresh and cryopreservation controls under vitrification and slow freezing protocols.

Groups	Number of primordial follicles			Number of primary follicles		
	Total	Normal	Percentage	Total	Normal	Percentage
Fresh	208	168	80.95 ± 1.34	53	33	62.54 ± 1.56
Slow Freezing-Control	96	68	71.45 ± 2.10^a^	37	18	49.66 ± 2.97^b^
Slow Freezing-Melatonin	127	94	74.30 ± 2.66	46	35	54.71 ± 2.86
Slow Freezing-BAPTA	114	83	72.95 ± 2.86	34	19	55.61 ± 2.37
Slow Freezing-Melatonin + BAPTA	105	75	72.50 ± 2.84	39	20	52.06 ± 2.34^a^
Vitrification-Control	112	76	68.15 ± 2.76^c^	43	22	51.12 ± 2.87^a^
Vitrification-Melatonin	121	92	76.35 ± 2.08	38	22	57.91 ± 2.73
Vitrification-BAPTA	137	105	77.45 ± 1.93	50	30	59.31 ± 2.51
Vitrification-Melatonin + BAPTA	87	64	74.25 ± 2.11	28	15	55.61 ± 2.62

^a^*p* < 0.05 vs. fresh group.

^b^*p* ≤ 0.01 vs. fresh group.

^c^*p* ≤ 0.001 vs. fresh group.

### TAC/ROS ratio

The TAC/ROS ratios in all groups and their related p-values are presented in Fig. 1D. The TAC/ROS ratio is significantly reduced from one in the fresh control group to 0.35 ± 0.02 (*p* < 0.001) and 0.39 ± 0.04 (*p* < 0.001) in the control slow freezing and vitrification groups, respectively. Also, in the slow freezing groups, the TAC/ROS ratio increased significantly to 0.62 ± 0.02 (*p* < 0.001), 0.56 ± 0.04 (*p* = 0.001), and 0.55 ± 0.05 (*p* = 0.003) in melatonin, BAPTA-AM and melatonin + BAPTA-AM supplemented cryopreservation groups, respectively compared to control slow freezing group. Vitrified groups supplemented with melatonin, BAPTA-AM, and melatonin + BAPTA-AM showed significantly increased the TAC/ROS ratio compared to the control freezing group and reached 0.67 ± 0.04 (*p* < 0.001), 0.62 ± 0.03 (*p* < 0.001), and 0.57 ± 0.04 (*p* = 0.010), respectively. Also, there were no significant differences in TAC/ROS ratio in vitrification control and treatment groups compared to their levels in counterpart slow freezing groups. Our results demonstrated that the correlation between ovarian tissue cells viability and the TAC/ROS ratio was significant and higher (r = 0.639, *p* < 0.001) than its correlation with ROS (r =  − 0.198, *P* = 0.048) and TAC (r = 0.423, *p* < 0.001). Also, the number of viable follicles showed a significant correlation with the TAC/ROS ratio (r = 0.287, *p* = 0.011), while it was not correlated with ROS levels (r = − 0.135, *p* = 0.146) and total antioxidant levels (r = 0.123, *p* = 0.329).

## Discussion

Over the last few decades, OTCT has been developed as an effective method to preserve fertility in female cancer survivors^44,45^. However, there are concerns regarding the risk of reintroducing malignant cells after reimplantation of ovarian tissue in girls and women with blood-born leukemias or cancers with a high risk of ovarian metastasis. In these patients OTC can preserve ovarian tissue with the hope of further development of the current experimental techniques such as in vitro maturation of oocytes, artificial ovary, stem cell-based oogenesis, etc^46,47^. Moreover, OTCT is associated with large-scale follicle loss and short graft longevity after transplantation^7^. Therefore, much remains to be done to optimize ovarian tissue cryopreservation methods. Ca^2+^ plays a significant role as a secondary messenger in pathways that influence ROS generation and cell death^48^. On the other hand, a recent study in oocytes found that ethylene glycol (EG) and dimethyl sulfoxide (DMSO), which are routinely used as a permeable CPA in ovarian tissue cryopreservation solution, cause Ca^2+^ overload in the cytosol^27^. To the best of our knowledge, the role of intracellular Ca^2+^ during ovarian tissue cryopreservation is largely unknown. This study is the first to explore the effect of BAPTA-AM as a calcium ion chelator on ovarian tissue cells and follicle viability, and oxidative status during cryopreservation using both slow freezing and vitrification methods. Also, we evaluated the synergistic effects of BAPTA-AM and melatonin on the cryopreservation of bovine ovarian tissue. Our results showed that BAPTA-AM can protect ovarian tissue cells against cryopreservation-induced damage by reducing ROS generation and cell death, likely by improving the antioxidant defense system. These positive effects are comparable to the protective effect of melatonin on frozen-thawed ovarian tissue.

Our results indicated that cryopreservation significantly reduced total cell viability of ovarian tissues in both vitrification and slow freezing methods compared to the fresh control group, and supplementation of cryopreservation media with BAPTA-AM or melatonin significantly improved total cells viability in both slow freezing and vitrification methods compared to their non-treated control freezing groups. In line with our findings, Wu et al. reported that the addition of 50 and 100 μM BAPTA-AM to sperm freezing media caused higher viability and motility in cryopreserved sperm compared with the non-treated freezing group^21^. Another study reported that the calcium chelator BAPTA-AM was able to significantly improve the viability of cells and inhibit iron overload-induced cell apoptosis in chondrocytes^49^. One possible mechanism responsible for cell death during cryopreservation could be the overload of intracellular Ca^2+^ in the cytosol induced by exposure to permeable CPAs such as EG and DMSO^24^. This intracellular Ca^2+^ overload might trigger the opening of the mitochondrial permeability transition pores (mPTP), leading to ROS and Ca^2+^ discharge, mitochondrial membrane potential (∆Ψm) impairment, decreased adenosine triphosphate (ATP) levels, and the release of cytochrome C. These events can cause DNA damage and cell death^24^. Another explanation is that osmotic stress induced intracellular Ca^2+^ overload, may stimulate nicotinamide adenine dinucleotide phosphate oxidase (NADPH oxidase) activity and ROS production through activation of phospholipase A2 and, again, cause cell death^50^. Also, during cryopreservation ROS generation can induce cell death by activating of caspases 3, 8, and 9^51^, or via the ROS-JNK-p53 pathway, which is associated with an increase in apoptotic molecules such as Bax, Bak, and Puma, and a decrease in anti-apoptotic molecules such as Bcl-2, Bcl-xL, and Mcl-1^52^. Both Ca^2+^ and ROS are important bidirectional secondary cell signaling messengers^35^. According to these abovementioned mechanisms, our results suggest that BAPTA-AM possibly increases ovarian tissue cells viability during cryopreservation through modulation of intracellular calcium levels with its subsequent ROS generation.

Previous research supports our results on the beneficial effect of melatonin on frozen-thawed cells and tissue viability and the inhibitory effect of melatonin in cell apoptosis^29,32,53^. Moreover, our results revealed that BAPTA-AM had a similar effect on frozen-thawed ovarian tissue cells viability compared to melatonin in both slow freezing and vitrification procedures. Interestingly, when we supplemented freezing media with the combination of BAPTA-AM and melatonin, the ovarian tissue cells viability decreased compared to the individually supplemented melatonin or BAPTA-AM freezing groups. This might be a result of reductive stress, which is the counterpart to oxidative stress, where oxidants are reduced at the physiological level, and may be caused by excess amounts of glutathione^54^ or overexpression of available antioxidant proteins^55,56^. On a cellular level, the presence of increased reducing equivalents and the lack of beneficial levels of ROS can prevent growth factor-dependent signaling pathways, promote mitochondrial dysfunction, increase apoptosis, and reduced cell survival^57^. Based on our finding, ovarian tissue cells viability (total cells viability) was higher in vitrified groups compared to slow freezing groups. Because stromal cells are the main population of ovarian cortex (80% cell population), these confirm previous findings that indicated slow freezing induces more damage to ovarian stromal cells compared to vitrification^58^.

In the case of viable follicles, our results indicated no significant difference in the number of viable follicles between frozen-thawed tissues and the fresh group in slow freezing techniques, while significant reductions were observed in the number of viable follicles in vitrified ovarian tissue (non-treated group) compared to fresh group. In line with our finding, Oktem et al. showed that slow freezing can preserve human primordial ovarian follicles better than the vitrification method in frozen-thawed ovarian tissue^59^. On the other hand, Silber et al. reported superiority of vitrification method when they compared the viable oocytes in vitrified tissue and slow freezing tissue^60^. In contrast, other studies failed to find significant differences between the two procedures in the case of viable and apoptotic follicles^61–63^. This controversy might be related to the size of the cortical pieces, or the methods chosen in different studies for the evaluation of follicles viability within the tissue. Considering that follicles distribute heterogeneously within the ovarian cortex means that some sample bias happens because of the follicle density difference in cortical tissues. Note that there are various techniques to examine ovarian tissue viability in the literature^63–65^, but a standard protocol remains to be identified. Finally, our finding is in line with previous findings indicating increased susceptibility of stromal cells to cryopreservation induced damage when compared to follicles^10,66^.

Our data suggest that ovarian tissue ROS generation is higher in both vitrification and slow-freezing groups compared to the fresh group. This is in agreements with previous studies^12,67^. Xu et al. reported that exposure of human embryonic stem cells (HESCs) to DMSO without freezing (addition and removal of DMSO), resulted in doubled O_2_^•−^ levels. Also, they reported during cryopreservation of HESCs, in the presence of the same amount of DMSO, the O_2_^•−^ levels increased five times, which confirmed that the stress from freezing and thawing can enhance ROS generation^68^. Interestingly, when cryopreservation media have been supplemented with ice nucleation inhibitors, such as anti-freeze proteins (AFP) and polyethylene glycol (PEG), ROS levels have been decreased in frozen-thawed cells possibly through reducing cryo-injury by stabilizing mitochondrial function and protecting the glutathione (GSH) synthesis-related proteins during freezing and thawing^69,70^. ROS generation during cryopreservation is related to osmotic stress in cells and possibly acts as a signaling pathway responsible for the cell adaptive reaction^35,71^. It is also believed that frozen thawed cells generate more energy to repair cell structural damage, resulting in higher ROS production^72,73^. Intracellular ROS is mainly sourced from the mitochondrial respiratory chain enzyme complexes and plasma membrane NADPH oxidase along with other enzymes in the cytosol, ER, and peroxisomes^74^. It is a widely held view that mitochondrial ROS regulate ROS production by non-mitochondrial sources. However, the precise impact of mitochondria on total ROS generation in the cells is not fully understood^75^. Furthermore, we found that ROS levels are higher in the vitrification group than in the slow freezing group. Similarly, Somoskoi et al. reported that both slow freezing and vitrification techniques cause alteration in mitochondrial distribution and ROS production in mouse embryos and ROS production is higher in the vitrification method compared to the slow freezing at morula stage embryos^76^.

Along with the previously mentioned possible mechanism for ROS generation through the overload of calcium ions in the cells, another mechanism could be the alteration of the arrangement and/or abundance of proteins responsible for scavenging ROS and antioxidant enzymes such as glutathione peroxidase (GSH-Px), glutathione reductase (GR) and superoxide dismutase (SOD)^77^. Additionally, it is believed that free radicals attack polyunsaturated phospholipids, resulting in the formation of malondialdehyde (MDA), 4-hydroxynonenal (4HNE), and other toxic by-products, which can spread out into other cellular organelles and attach to protein nucleophiles, forming adducted proteins^48,78^. Adduct formation decreases and even disrupts protein function after altering protein structure. Aitken et al. showed that axonemal proteins, such as dyneins, tubulins, and heat-shock proteins, as well as mitochondrial proteins, like succinate dehydrogenase and ATP synthases, are common targets of these lipid peroxides^54^. Protein adduction in the first case disturbs cytoskeleton structure, while in the second case, it adversely affects the activity of the electron transport chain. The electron transport chain is an essential component of mitochondrial oxidative phosphorylation involved in ATP production. Consequently, disturbances in the electron transport chain due to protein adduction leads to ATP depletion and a self-perpetuating cycle of ROS production that eventually initiates the apoptotic cascade^48^. Additionally, our study found that when cryopreservation media were supplemented with a calcium chelator and/or melatonin, the levels of ROS were reduced considerably in treatment groups compared to the control freezing group. Like our finding, Sun et al. showed that the calcium chelator BAPTA-AM could reduce necroptotic characteristics such as mitochondrial ROS promotion and membrane depolarization in human colon cancer cells^79^. Other studies indicated that BAPTA-AM can reduce ROS levels in human alveolar epithelial cells^80^ and hydrogen peroxide (H_2_O_2_) levels in human keratinocyte cells^23^. Moreover, in agreement with our finding, previous studies showed the ameliorative effect of melatonin in cryopreserved induced ROS levels in different cells and tissues^12,32^. Considering lower-level ROS production in frozen-thawed ovarian tissues when we supplemented freezing media with a combination of BAPTA-AM and melatonin compared to supplemented groups with BAPTA-AM or melatonin individually, this might be a result of the different signaling pathways that BAPTA-AM and melatonin induced their effects. It seems that BAPTA-AM might reduce ROS levels through control of intracellular Ca^2+^ overload in the cells' cytosol as previously mentioned, and melatonin causes its effect by directly scavenging ROS^81^ and inhibiting the NADPH oxidase assembly and activity^82^. It is clear that both molecules directly and indirectly target sources of ROS production such as the mitochondrial respiratory chain enzyme complexes, plasma membrane NADPH oxidase, etc^83,84^.

Our results demonstrated that cryopreservation induced the reduction in total antioxidant levels in both freezing techniques compared to the fresh control group. TAC levels were increased in melatonin and BAPTA-AM treatment groups in the slow freezing procedure. In the vitrification groups, TAC levels increased in the melatonin group, while there were no significant differences between TAC levels in BAPTA-AM group and control vitrified group. While this study reveals the antioxidant effect of BAPTA-AM, the antioxidant potency of BAPTA-AM is less powerful than melatonin. In line with our finding about the antioxidant capacity of BAPTA-AM, a previous study in Madin-Darby Canine Kidney cells showed that BAPTA-AM-nanoparticles can increase SOD activity and restore MDA levels and can decrease the Bax/Bcl-2 ratio, the caspase 3 activity, and the release of cytochrome C, as well as the number of TUNEL-positive apoptotic cells. This demonstrates that BAPTA-AM plays a renal-protective role, probably through antiapoptotic and antioxidant mechanisms^85^. Another study indicated that the BAPTA-AM-loaded liposomes induce hepatoprotective effects through the increased activity of intracellular antioxidant enzymes, such as GSH, GSH-Px, and SOD, and reduced MDA levels. This study reported that BAPTA-AM-loaded liposomes could reduce oxidative stress, restrict TNF-α receptor, and mitochondria-mediated apoptotic pathways followed by a reduction of overloaded intercellular calcium^86^. In the case of melatonin, there is a large body of evidence in the literature that supports the effective role of melatonin in increasing the activity of intracellular antioxidant enzymes in different cells^13,30,31^. For instance, Feng et al. showed that melatonin significantly enhanced the protein expression and activity of SOD, catalase (CAT), and GSH-Px, which are the main antioxidant proteins in cells and are responsible for converting superoxide radical into water (SOD1 and SOD2: dismutase convert the superoxide radical into H_2_O_2_ and oxygen, and then GSH-Px and CAT enzymes convert the hydrogen peroxide to water)^32^.

In the case of percentage of morphologically normal follicles in frozen-thawed ovarian tissues, this study showed that while slow freezing and vitrification can protect primordial and primary follicles similarly, both methods cause a significant reduction in the percentages of intact follicles compared to the fresh group. There is a controversy in the literature about the effects of slow freezing and vitrification approaches on morphology of the ovarian follicles. In line with our findings, many studies reported similar findings in terms of follicular quality between slow freezing and vitrification methods and compared to fresh sample^87–90^. However, some studies reported the superiority of one method over the other method^59,60^, or their ability to preserve follicular morphology similar to fresh group^43^. This controversy might be related to CPA type and concentration, cryo device type and sample size and source.

According to our results, the ROS and TAC levels on their own may not perfectly capture ovarian tissue or follicle viability in frozen-thawed ovarian tissue. Here we presented a new metric to measure oxidative stress (TAC/ROS ratio), which showed a more powerful correlation with ovarian cortical cell/follicle viability in cryopreserved ovarian tissue compared to ROS or TAC levels alone both in vitrification and slow-freezing techniques. Also, our results indicated that the TAC/ROS ratio was significantly reduced in the frozen-thawed ovarian tissue compared to the control fresh group. In both techniques, this value was increased in melatonin and/or BAPTA-AM supplemented groups compared to the control freezing groups. In line with this finding, a previous study found that a reduction in sperm motility and viability is associated with a decrease in the ROS/TAC score (oxidative stress status)^91^. Agarwal et al. suggested the ROS/antioxidant score to measure oxidative stress in human seminal fluid and showed its correlation with infertility in men with a male factor or idiopathic diagnoses^92^. Finally, another study showed that ROS/TAC scores were significantly reduced in varicocele patients compared to healthy men^93^.

## Conclusions

In conclusion, our study illustrates that treatment of freezing media with BAPTA-AM could reduce the negative effect of cryopreservation on frozen-thawed ovarian tissues in both slow freezing and vitrification techniques. BAPTA-AM possibly induced its protective effects by modulating the intracellular Ca^2+^ level. Also, these protective effects of BAPTA-AM were comparable with the melatonin effect on frozen-thawed ovarian tissue. We also found that the TAC/ROS ratio predicts better ovarian tissue cells/follicles viability after cryopreservation compared to ROS or TAC levels alone. Our results will help to illustrate the approaches to modulate intracellular Ca^2+^ levels during cryopreservation that may help to optimize current cryopreservation protocols for clinical application.

## Methods

### Chemicals

All chemicals were obtained from the Thermo Fisher Company (Mississauga, ON, Canada) unless otherwise stated.

### Ovary collection and processing

Animal ethics approval was acquired from the University of Saskatchewan University Animal Care Committee (UACC; #024Exempt2021), all experiments were performed in accordance with UACC guidelines and have been reported using ARRIVE 2.0 guidelines. Pairs of whole ovaries were taken from 14- to 18-month-old bovines, at a local abattoir (n = 20). Under a laminar flow hood, the ovaries were washed once with 75% alcohol and then three times in the fresh MEM medium supplemented with 1% streptomycin-penicillin G and 25 mmol/L HEPES. After the separation of the medulla with a scalpel and surgical scissors, the ovarian cortex was cut into small pieces of 5 × 5 × 1 mm^94,95^. Then, 55 ± 3/animal of equally sized cortical fragments (5 × 5 × 1 mm) were selected from each pair of ovaries and were randomly allocated to 9 different groups, including a fresh control group (6 pieces), four slow freezing groups (24 pieces), and four vitrification groups (24 pieces). From each group/animal, one piece was used for ovarian tissues cells viability measurement, one piece was utilized for ROS and TAC assessment, two pieces were employed for follicular viability evaluation and two pieces were used for follicular morphology assessment.

### Slow freezing

For slow freezing experiments, cortical tissue fragments from each animal were randomly allocated into 4 slow freezing experimental groups. Four different freezing solutions were used: (1) the base slow freezing medium (Leibovitz medium supplemented with 1.5 mol/L DMSO, 0.1 mol/L sucrose and 10% human serum albumin (control freeze group)), (2) the base slow freezing medium + melatonin (0.1 mmol/L), (3) the base slow freezing medium + BAPTA-AM (50 µmol/L), (4) the base slow freezing medium + BAPTA-AM (50 µmol/L) + melatonin (0.1 mmol/L)^36,96^. 1.5 mL cryovials containing one of the above mentioned cryopreservation solutions and ovarian fragments were immediately placed in a pre-cooled programmable controlled rate freezer (ThermoScientific, 7456) with the following program: (1) CPA equilibration at 4 °C for 30 min; (2) cooling to − 7 °C at − 2 °C/min; (3) maintenance at − 7 °C for 10 min, where manual seeding was performed by touching the side of each cryovial with a forceps cooled in liquid nitrogen until the ice was observed to nucleate in the vial; (3) cooling to − 40 °C at − 0.3 °C/min; (4) cooling to − 140 C at − 10 °C/min^36,96^; (4) plunging directly into the liquid nitrogen and storage in a liquid nitrogen dewar for future use.

### Vitrification

Cortical tissue fragments from each animal were randomly divided into four groups in the vitrification procedure: (1) the base vitrification solution (Ova Cryo Kit, Kitazato; referred to as the control vitrification group); (2) the base vitrification solution + BAPTA-AM; (3) the base vitrification solution + melatonin; (4) the base vitrification solution + BAPTA-AM + melatonin. The ovarian tissue was cryopreserved using a vitrification kit (Ova Cryo Kit Type M, VT-301S, Kitazato Corporation) according to the manufacturer’s protocol. Briefly, the ovarian cortexes were sequentially immersed in the Kitazato Kit’s Cryo1 solution (+ /- supplements), Cryo2 solution (+/− supplements), and Cryo3 solution (+/− supplements) for 5, 5, and 15 min, respectively. All steps were performed at room temperature per kit instructions. Then, the tissues were loaded into the precooled closed ovarian storage device (Ova tube, Kitazato, Shizuoka, Japan) and plunged directly into the liquid nitrogen, and transferred to a storage dewar for future use.

### Thawing

The cryovials from all slow freezing groups were thawed at room temperature (between 21 and 23 °C) for 2 min and then were immersed in a water bath at 37 °C for another 2 min^36,37^. Vitrified ovarian tissues were thawed with a thawing kit (Ova Thawing Kit Type M, VT- 302S, Kitazato Corporation, Shizuoka, Japan) according to the manufacturer's protocol. Briefly, ovarian tissues were placed into the prewarmed Thawing 1 solution for 1 min at 37 °C. After detaching the ovarian tissue from the device, the ovarian tissues were transferred to Thaw 2 solution for 3 min and then Thaw 3 solution for 5 min at room temperature. Previous studies have shown that a minimum 60 min washing step is necessary after ovarian tissue cryopreservation to minimize the cryoprotectant toxicity^97^. In this study, after removing the cryoprotectant with fresh medium (TCM-199), ovarian tissues were put into maintenance culture media (TCM-199 supplemented with L-glutamine, sodium bicarbonate 1% streptomycin-penicillin G and 10% fetal bovine serum (FBS)) at 37 °C in 5% CO_2_ for 2 h before further processing^98^. Fresh ovarian fragments were also cultured in the same maintenance media at 37 °C in 5% CO_2_ for 2 h.

### Ovarian tissues cells viability measurement

Calcein-AM staining was performed to measure the viability of frozen-thawed ovarian tissue according to the Luckenbach et al. method with some modifications^99^. Briefly, tissue fragments were incubated with 2 μM calcein-AM for 45 min at 37 °C and 5% CO_2_. The cortical fragment was then rinsed and washed with a cold phosphate-buffered saline (PBS). Cortical fragments were then homogenized in ice cold ddH_2_O supplemented with 50 mmol/L Tris–HCl, pH 7.5 (1/10, w/v). Tissue lysates were spun at 3000 g for 10 min to remove the particles. Then 100 µL of the obtained supernatants of each sample were transferred to 96 well plate. Calcein fluorescence intensity was then quantified using a plate reader (Varioskan Flash) at an emission wavelength of 530 nm. Each sample was tested in duplicate. As a negative control for calcein-AM, the tissue was exposed to 3% formaldehyde at 4 °C for 30 min to induce cell death and then subjected to the same staining and homogenization procedures. To assess the background fluorescence of the dye, calcein-AM was added to a subset of wells without supernatant. All fluorescence procedures were performed in a darkroom. For each experiment, fluorometric measurements were expressed as fluorescence intensity units (FIU).

### Reactive oxygen species levels measurement

Tissue ROS levels were measured using 2′,7′-Dichlorofluorescein diacetate (DCFH-DA; Sigma-Aldrich, Oakville, ON, Canada; 287810). Briefly, cortical fragments were washed with cold phosphate-buffered saline (PBS). Cortical fragments were then homogenized in ice cold ddH_2_O supplemented with 50 mmol/L Tris–HCl, pH 7.5 (1/10, w/v). Tissue lysates were spun at 3000 g for 10 min to remove the particles. Then 100 µL of the supernatant was incubated with DCFH-DA (25 µmol/L) for 20 min. The formation of fluorescent dichlorofluorescein as the product of DCFH-DA oxidation in the presence of ROS was monitored with a fluorescence microplate reader (Varioskan Flash) at 488 nm excitation and 525 nm emission The non-stained aliquot of each sample was used as a negative control to determine their auto-fluorescence background. The lysate of H_2_O_2_-treated tissues was used as a positive control. To assess the background fluorescence of the dye, DCFH-DA was added to a subset of supernatant-free wells. All fluorescence procedures were performed in a darkroom^100^. For each experiment, fluorometric measurements were performed in duplicate and expressed as fluorescence intensity units (FIU).

### Total antioxidant capacity measurement

To detect the tissue's total antioxidant capacity (TAC), the TAC Assay Kit (Abcam, Cambridge, UK; ab65329) was used according to the manufacturer's protocol. Briefly, TAC was assessed after homogenizing tissue fragments in a cold ice ddH_2_O supplemented with 50 mmol/L Tris–HCl, pH 7.5 (1/10, w/v). Tissue lysates were spun at 3000 g for 10 min to remove the particles. Then 100 µL of the Supernatants from each sample were incubated with the Cu^2+^ working solution for 90 min on a shaker at room temperature. The small molecule and protein antioxidants convert Cu^2+^ ions to Cu^+^. Absorbance was detected using a microplate reader (Varioskan Flash) at 570 nm. TAC was calculated using a standard curve, and results were expressed as antioxidant concentration (mmol). Each sample was tested in duplicate. Trolox (6-hydroxy-2,5,7,8-tetramethyl chroman-2-carboxylic acid) (0, 12, 24, 36, 48, 60 μL) was used as a standard to determine the Trolox equivalent capacity of the tested samples.

### Total protein content quantification

The protein levels of each sample were measured using a BCA protein assay kit according to the manufacturer's protocol (Sigma-Aldrich, Oakville, ON, Canada; B9643). The principle of the bicinchoninic acid (BCA) assay is the formation of a Cu^2+^ -protein complex under alkaline conditions, followed by the reduction of the Cu^2+^ to Cu^1+^. 200 µL of the BCA Working Reagent are mixed with 25 µL of a protein sample in 96 well plates and incubated at 37 °C for 30 min. Absorbance was detected using a microplate reader (Varioskan Flash) at 562 nm. The sample was either a blank, a BSA protein standard, or an unknown sample. The blank consisted of a buffer with no protein. The BSA protein standard consisted of a known concentration of bovine serum albumin (ranging between 200 and 1000 mg/ml), and the unknown sample was the solution to be assayed. The amount of reduction is proportional to the protein present. Then, the obtained values were employed to correct viability, ROS, and TAC test results. For each sample, protein activity was normalized to its protein concentration. All samples were run in duplicate.

### Assessment of follicular viability

Follicle viability was evaluated by calcein-AM (Invitrogen, Carlsbad, CA) staining, using a protocol published previously with some modifications^15,63^. Ovarian cortical fragments were transferred to 1 mL of TCM-199 medium supplemented with 2 µmol/L calcein-AM and incubated for 1 h at 37 °C in 5% CO_2_. Then, the fragments were placed in 2 µmol/L calcein AM/DPBS solution supplemented with Liberase DH enzyme (0.08 mg/mL) (Roche Diagnostics, Mannheim, Germany) and 1% streptomycin-penicillin G and incubated for 45 min at 37 °C in 5% CO_2_. Gentle pipetting was also done every 15 min. The reaction was ended by the addition of cold DPBS supplemented with 10% FBS at room temperature. Calcein-AM is cleaved by intracellular esterase enzymes in viable cells. Follicles less than 80 µm (primordial and primary) were classified as viable if the oocyte and its surrounding granulosa cells were stained with calcein-AM (green)^101^. Due to the non-uniform distribution of follicles within the ovarian cortex, 15–130 follicles in all fields were evaluated for each ovarian piece^102^. Viable follicles were detected using fluorescence microscopy (Nikon, Eclipse80i, Tokyo, Japan). The calcein-AM excitation/emission wavelengths were 485/530 nm.

### Tissue histology evaluation

Ovarian cortical fragments from all experimental groups were fixed and dehydrated in 4% paraformaldehyde (4 °C overnight) and ascending ethanol concentrations (50, 70, 80, 90, and 100%). The fixed tissues were embedded in paraffin wax, and 5 µm-thick tissue sections (every 10th section) were mounted on a glass slide. After deparaffination and hydration in a descending gradient of ethanol, the slides were stained with hematoxylin–eosin (H & E). Then tissue sections were dehydrated in ascending ethanol, cleared in xylene, and mounted with a mounting medium and a coverslip. Based on the granulosa cell morphology and the number of layers surrounding the oocytes, the developmental stages of the follicles were classified into the following categories: primordial follicle, a follicle with the oocyte surrounded by a layer of flattened granulosa cells; primary follicle, a follicle with the oocyte surrounded by a complete layer of cuboidal granulosa cells^61^. Morphologically normal follicles were identified as those showing a uniform distribution of granulosa cells, intact basal membrane, and a round oocyte with a uniform cytoplasm and nucleus with normally dispersed chromatin. Follicles were classified as damaged if there was at least one of the following signs: detachment of the granulosa cells from the basement membrane as well as from neighboring granulosa cells, detachment of the oocyte from surrounding granulosa cells, the shrinkage of the oocyte, vacuolization in the oocyte, a granulosa cell or an oocyte with a pyknotic nuclei. Follicular morphology was evaluated under a light microscope (Nikon, Tokyo, Japan) with a magnification of ×400. To avoid double counting, only follicles displaying oocytes were counted.

### TAC/ROS ratio

Several indexes have been suggested to measure oxidative stress in humans and its relationship with health and disease status including Oxidative Stress Index (OSI), Tiol Ratios (-SH/TT, -SS/-SH, and-SS/TT), glutathione ratio (GSSG/GSH), oxidative stress score (OSS). Here we suggest the normalized total antioxidant capacity proportion on the normalized ROS level. The ROS and TAC values of the fresh (control) tissue from each animal were used to normalize these values from the same animal in treated groups in both slow freezing and vitrification groups. Then the ratios of normalized total antioxidant/normalized ROS levels were used to measure oxidative stress status. This ratio is unit valued in fresh control groups and the deviation of this value from one to zero presents a higher TAC/ROS imbalance. Then the correlation between ROS, TAC, and TAC/ROS ratio with frozen-thawed ovarian tissue cells and follicle viability in all cryopreserved groups was evaluated both in slow freezing and vitrification procedures^103,104^.

### Statistical analysis

SPSS software (version 23) was used to analyze the data. The data were tested for normality by using the Kolmogorov–Smirnov test. Statistical analysis was carried out using the two-way analysis of variance (ANOVA) with “group” and “cryopreservation method” as independent variables. Following significant “group” × “cryopreservation” interaction, multiple comparisons were performed using Tukey’s post hoc test. Correlation analysis was conducted using Pearson’s r tests. The results are presented as the mean ± standard error or median (IQR) as appropriate. In this study, statistical significance was set at *p* < 0.05.

## Data Availability

The datasets generated during and/or analyzed during the current study are available from the corresponding author on reasonable request.

## Abbreviations

OTCT: Ovarian tissue cryopreservation and transplantation
OTC: Ovarian tissue cryopreservation
ROS: Reactive oxygen species
TAC: Total antioxidant capacity
POF: Premature ovarian failure
CPAs: Cryoprotectant agents
PUFAs: Polyunsaturated fatty acids
EGTA: Ethylene glycol tetra acetic acid
BAPTA-AM: 1,2-Bis (*o*-amino phenoxy) ethane-*N*,*N*,*N*′,*N*′-tetra acetic acid
IVF: In vitro fertilization
DMSO: Dimethyl sulfoxide
HEPES: (4-(2-Hydroxyethyl)-1-piperazineethanesulfonic acid)
DCFH-DA: 2′,7′-Dichlorofluorescein diacetate
FIU: Fluorescence intensity units
BCA: Bicinchoninic acid
OSI: Oxidative stress index
OSS: Oxidative stress score
ATP: Adenosine triphosphate
AFP: Anti-freeze proteins
PEG: Polyethylene glycol
GPx: Glutathione peroxidase
GR: Glutathione reductase
MDA: Malondialdehyde
4HNE: 4-Hydroxynonenal
